# A bibliometric analysis of research on craniomaxillofacial distraction osteogenesis from 2000 to 2021

**DOI:** 10.3389/fsurg.2022.932164

**Published:** 2022-08-01

**Authors:** Zhen Liu, Jianying Yang, Changhan Zhou, Yao Liu, En Luo

**Affiliations:** ^1^ State Key Laboratory of Oral Diseases & National Clinical Research Center for Oral Diseases, West China Hospital of Stomatology, Sichuan University, Chengdu, China; ^2^ Department of Outpatient nursing, West China Second University Hospital, Sichuan University, Chengdu, China; ^3^ Key Laboratory of Birth Defects and Related Diseases of Women and Children (Sichuan University), Ministry of Education, Chengdu, China

**Keywords:** bibliometrics, CiteSpace, distraction osteogenesis, craniomaxillofacial dysplasia, bone defects, orthognathic surgery

## Abstract

**Objective:**

This study collected and summarized publications related to craniomaxillofacial distraction osteogenesis(DO) from 2000 to 2021, investigated trends in related research, and compared publications from different countries, institutions and journals. The aim is showcasing hotspots and frontiers in the field and providing a reference for future research.

**Background:**

Craniomaxillofacial DO serves to treat different types of craniomaxillofacial dysplasia and bone defects and deformities. DO can significantly reduce surgical trauma, complications, and recurrence rate compared to conventional surgery. However, there is a lack of bibliometric analyses regarding Craniomaxillofacial DO.

**Methods:**

CiteSpace and VOSviewer were used to analyze and visualize 3,141 articles and reviews searching through the Web of Science Core Collection(WOSCC) to obtain publications on craniomaxillofacial DO from 1 January 2000 to 31 December 2021.

**Results:**

In the last 21 years, there has been a significant increase in the number of publications. The United States, the People's Republic of China, and Italy produce the vast majority of publications. University of Milan and University of Bologna are the most influential in this field. McCarthy JG is the most influential author. Obstructive sleep apnea, TMJ ankylosis and cleft lip and palate are potential research direction in this field.

**Conclusion:**

Future research should focus on the precise indications and optimal timing of craniomaxillofacial DO and the evaluation of the long-term outcomes of various modified procedures. This study provides a relatively objective reference for related researchers, medical practitioners, and global health systems.

## Introduction

Distraction osteogenesis (DO) is a surgical procedure for correcting skeletal deficiencies or repair bone defects by applying a specific amount of traction or expansion force to an incised bone segment to regenerate new bone in the interstitial space to lengthen or widen the bone (1). The principle of DO can be explained by the “law of tension-stress” proposed by Ilizarov, which states that the progressive application of a continuous tension force to living biological tissue stimulates and maintains its tissue regeneration and growth (1). DO was initially applied to the long bones of the limbs and was successfully applied clinically in the craniomaxillofacial treatment by McCarthy in 1992 (2). So far, DO has been a commonly-used approach to treat craniomaxillofacial deformities.

Craniomaxillofacial DO, serves to treat different types of craniomaxillofacial dysplasia and bone defects and deformities. A wide range of indications for the treatment of various types of dysplasia and bone defects and deformities involving the craniomaxillofacial fields and their complications such as Crouzon Syndrome (3), craniosynostosis (4), vertical ridge deficiencies (5, 6), unilateral craniofacial microsomia (7) and so on. DO can significantly reduce surgical trauma, complications, and recurrence rates compared to conventional surgery, advancing the age at which patients can be orthodontically treated (8, 9).

Bibliometrics is a reliable tool for research assessment of publications to explore trends and frontiers on a certain research topic, such as cancer gene therapy (10), neck dissection for oral squamous-cell carcinoma (11) and so on. However, to the best of our knowledge, no one has yet conducted a bibliometric analysis of craniomaxillofacial DO so far. Thus, the current study aims to assess the research trends, country, institution, authors, journals, references and keywords of craniomaxillofacial DO-related publications to reveal the research frontiers and predict future trends for relevant researchers.

## Methods

### Data acquisition

**Figure 1:**
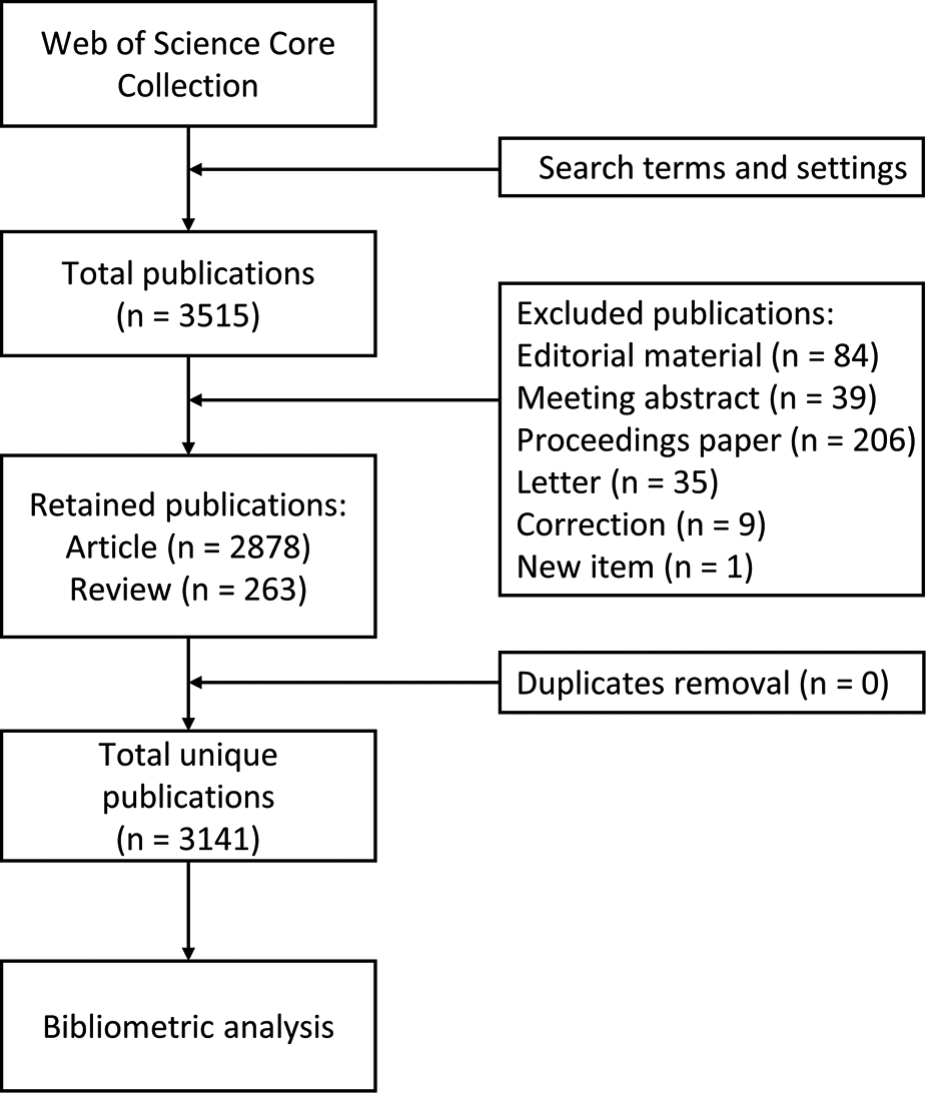
Process of literature search and filtration.

We conducted a systematic search of the Web of Science Core Collection for relevant literature to obtain publications on craniomaxillofacial DO from 1 January 2000 to 31 December 2021. We used the following search terms: TS = (distraction osteogenesis) AND TS = (maxillofacial OR craniofacial OR dentofacial OR maxillo-facial OR cranio-facial OR dento-facial OR maxilla* OR mandib* OR alveolar). The total number of publications was 3,515 after initial search. Then the publications were filtered by CiteSpace (Drexel University, Philadelphia, USA). Only articles and reviews were included, and other types of publications(editorial material, meeting abstract, proceedings paper, letter, correction and new item)as well as duplicates were excluded. Detailed information of the corrections were provided in Supplementary Appendix S1. Consequently, a total of 3,141 publications was included in the study (Figure 1).

### Analysis and statistics

We used CiteSpace to identify the keywords with burst and the references with burst. CiteSpace is software for analyzing and visualizing publication trends. Burst represents a dramatic increase in interest in the academic field. As a reflection of the academic trends and hot topics in a field at a given time, burst can help predict cutting-edge research directions (12). The visual maps consist of nodes and lines. The nodes represented elements including countries, institutions, authors, journals and keywords. The frequency of occurrence is represented by the size of the nodes. The co-occurrence or co-citation relationship is shown by the lines between the nodes. The nodes and lines' colors correspond to the average year of the relevant publication.

VOSviewer (Leiden University, Leiden, the Netherlands) was used to construct visualization maps of co-citation and co-occurrence analysis. In the clustering maps, different colors represent different clusters. There are differences in the themes represented by the different clusters. In other maps, VOSviewer applies different colors to the keywords based on different average years of occurrence. Elements in blue and green occur earlier, while the yellow and red ones appear later.

## Results

### Analysis of research trends

A total of 3,141 reviews and articles related to craniomaxillofacial DO were retrieved during the study period. The last two decades can be divided into two phases according to the research trends. The first phase lasted from 2000 to 2012, with a relatively rapid increase in the count of publications, including 208 publications in 2012 The second phase occurred from 2013 to 2021, with a relatively stable number of publications.

**Figure 2:**
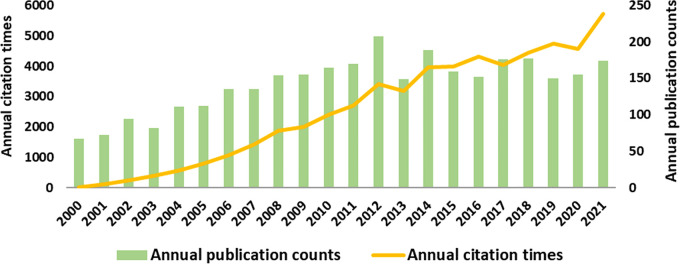
Analysis of global research trend of craniomaxillofacial distraction osteogenesis during 2000–2021.

From 2000 to 2021, the number of annual citation times showed a clear trend of increase, except for decreases in 2013, 2017 and 2020. Notably, there was a boost increase in annual citation times in 2021 (*n* = 5,735), compared to 2020(*n* = 4,572), indicating that craniomaxillofacial DO has recently attracted great academic attention from global researchers (Figure 2).

### Analysis of countries and institutions

**Figure 3:**
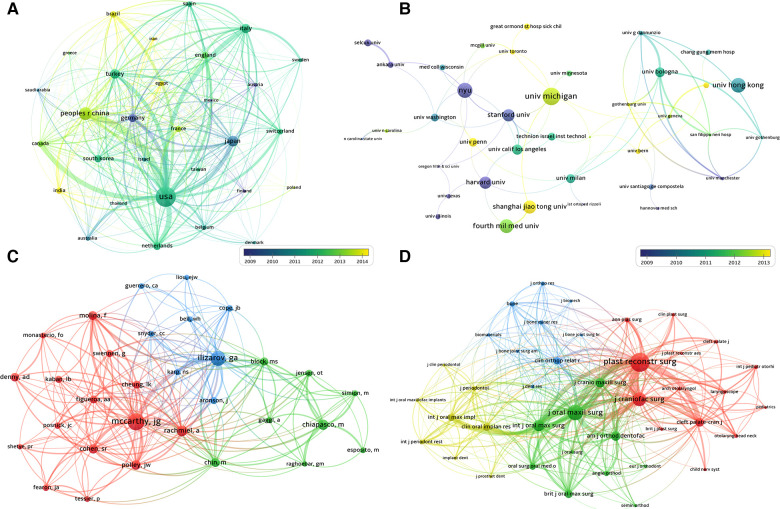
(**A**) Co-occurrence analysis of countries; (**B**) Co-occurrence analysis of institution; (**C**) clustering analysis of cited authors; (**D**) clustering analysis of cited journals.

As shown in Table 1, the United States had the most publications on craniomaxillofacial DO, with 958 publications, followed by People's Republic of China (*n* = 380), Japan (*n* = 261), Turkey (*n* = 218), Italy (*n* = 216) Germany (*n* = 157), Netherlands (*n* = 145), South Korea (*n* = 138), England(*n* = 106) and Brazil (*n* = 96). The co-occurrence analysis (Figure 3A) showed that the United States, People's Republic of China and Japan are the main research forces in craniomaxillofacial DO and a significant amount of cooperation is observed between the United States and other countries such as People's Republic of China, the Netherlands, Italy, Japan, Turkey and Canada. In addition, Germany and Japan have studied craniomaxillofacial DO for an earlier period, while Brazil, Egypt and India are later.

**Table 1:** The top 10 most productive authors and countries contributing to the research on craniomaxillofacial distraction osteogenesis from 2000 to 2021.

Rank	Author	Publication counts	Rank	Country	Publication counts
1	Buchman SR	57	1	USA	958
2	Cheung LK	48	2	PEOPLES R CHINA	380
3	McCarthy JG	41	3	JAPAN	261
4	Donneys A	38	4	TURKEY	218
5	Kaban LB	37	5	ITALY	216
6	Wolvius EB	35	6	GERMANY	157
7	Longaker MT	33	7	NETHERLANDS	145
8	Bartlett SP	32	8	SOUTH KOREA	138
8	Deshpande SS	32	9	ENGLAND	106
10	Taylor JA	31	10	BRAZIL	96

The top 5 institutions in terms of the number of relevant research publications are University of Michigan (with 72 publications), New York University (63), The University of Hong Kong (61), The Fourth Military Medical University (57) and Sichuan University (57). It is worth noting that among the top 5 institutions in terms of citation times, University of Milan (2695) and University of Bologna (1927) are not ranked in the top in terms of the number of publications, which indicates the greater influence of these two institutions in the craniomaxillofacial DO field (Table 2 and Figure 3B).

**Table 2:** The top 10 most productive and most cited institutions contributing to the research on craniomaxillofacial distraction osteogenesis from 2000 to 2021.

Rank	Institution	Publication counts	Rank	Institution	Citation times
1	Univ Michigan	72	1	Univ Milan	2,695
2	NYU	63	2	Univ Bologna	1,927
3	Univ Hong Kong	61	3	Univ Calif Los Angeles	1,715
4	Fourth Mil Med Univ	57	4	NYU	1,612
4	Sichuan Univ	57	5	Stanford Univ	1,490
6	Shanghai Jiao Tong Univ	53	6	Univ Manchester	1,478
7	Harvard Univ	50	7	Univ Hong Kong	1,322
8	Stanford Univ	49	8	Univ Michigan	1,233
8	Univ Calif Los Angeles	41	9	Univ Texas	1,196
10	Univ Bologna	39	10	Univ Bern	1,089

### Analysis of authors and journals

Table 1 shows the top 10 most productive authors contributing to the research on craniomaxillofacial DO from 2000 to 2021. The most active author in this field were Buchman SR(with 57 publications), Cheung LK(48) and McCarthy JG(41). McCarthy JG is the most influential author and has worked closely with Molina F and Cohen SR, Polley JW.

The top 10 most productive journals are listed in Table 3. The most productive journals are *Journal of Craniofacial Surgery* with 531 publications, followed by *Journal of Oral and Maxillofacial Surgery*(with 314 publications), *Journal of Cranio-Maxillofacial Surgery*(210), *International Journal of Oral and Maxillofacial Surgery*(207), *Plastic and Reconstructive Surgery*(185). Among the top 5 journals, *Plastic and Reconstructive Surgery* (with 6,343 citations, IF4.73), *Journal of Oral and Maxillofacial Surgery* (5,482, IF1.895) and *Journal of Craniofacial Surgery* (5,431, IF1.046) have the most total citations, which indicates that these three journals are the most influential and professional in the field of craniomaxillofacial DO. Scholars conducting research in this field can regard this as a reference to prioritize their articles for publication in these journals. Additionally, the analysis of Figure 3D shows that the most co-cited journals are *Craniofacial Surgery, Journal of Oral and Maxillofacial Surgery, Plastic and Reconstructive Surgery* and *Cleft Palate-craniofacial*, which reflects the similarity in publication themes of the three journals.

**Table 3:** The top 10 most productive journals contributing to the research on craniomaxillofacial distraction osteogenesis from 2000 to 2021.

Journal	Publication counts	Total citation times	Impact factor (2021)
*Journal of Craniofacial Surgery*	531	5,431	1.046
*Journal of Oral and Maxillofacial Surgery*	314	5,482	1.895
*Journal of Cranio-Maxillofacial Surgery*	210	2,908	2.078
*International Journal of Oral and Maxillofacial Surgery*	207	4,697	2.789
*Plastic and Reconstructive Surgery*	185	6,343	4.73
*British Journal of Oral & Maxillofacial Surgery*	112	1,472	1.651
*American Journal of Orthodontics and Dentofacial Orthopedics*	88	1,507	2.65
*Oral Surgery Oral Medicine Oral Pathology Oral Radiology*	58	1,245	2.589
*International Journal of Oral & Maxillofacial Implants*	57	3,373	2.804
*Clinical Oral Implants Research*	37	2,134	5.977

### Analysis of references

**Figure 4:**
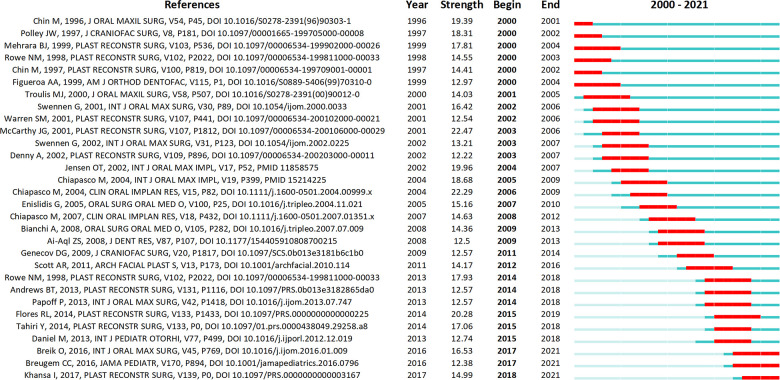
The top 30 cited references with the strongest burst.

The top 30 cited references with the strongest burst were present in Figure 4. The top 3 among them were as follows. “*Distraction osteogenesis of the craniofacial skeleton*” by McCarthy et al. (13) in 2001, which reviews the history, biology and clinical applications of DO; “Alveolar distraction osteogenesis vs. vertical guided bone regeneration for the correction of vertically deficient edentulous ridges: a 1–3-year prospective study on humans” by Chiapasco et al. (14) in 2004, which examined the ability of vertical directed bone regeneration with vertical DO to restore vertically deficient alveolar ridges. The results suggested that DO can reduce the incidence of complications and provide a more favorable long-term prognosis (14); “The surgical correction of Pierre Robin sequence(PRS): mandibular distraction osteogenesis vs. tongue-lip adhesion” by Flores et al. (15) in 2014. Flores et al. analyzed the outcomes of mandibular DO vs. tongue-lip adhesion in the surgical treatment of PRS, concluding that DO performs better in terms of improving airway function (15).

Notably, some recent publications were identified as references with burst, indicating that they are gaining much academic attention. Such publications are listed below: “*Mandibular distraction osteogenesis for the management of upper airway obstruction in children with micrognathia: a systematic review*.” by Breik et al. (16) in 2016; “*Best Practices for the Diagnosis and Evaluation of Infants With Robin Sequence: A Clinical Consensus Report.*” by Breugem et al. (17) in 2016; “*Airway and Feeding Outcomes of Mandibular Distraction, Tongue-Lip Adhesion, and Conservative Management in Pierre Robin Sequence: A Prospective Study.*” By Khansa et al. (18) in 2017.

The blue line represents the timeline and the red line represents the period in which a reference had a burst.

### Analysis of keywords

**Figure 5:**
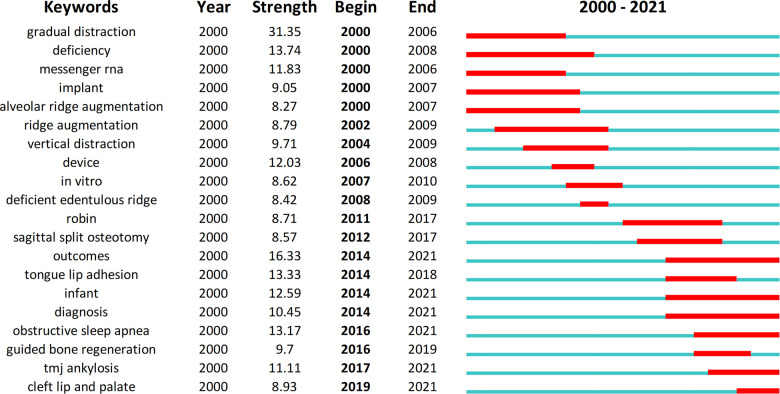
The top 20 keywords with the strongest burst.

The top 20 keywords with the strongest burst were shown in Figure 5. From 2000 to 2010, the keywords with the strongest burst included “gradual distraction”, “deficiency”, “messenger RNA”, “implant”, “alveolar ridge augmentation”, “ridge augmentation”, “vertical distraction”, “device”, “in vitro” and “deficient edentulous ridge”, which reflected the hotspots about the principle, operation of DO and its application in oral implantology and maxillofacial deficiency. Academic attention from 2011 to 2018 turned into the PRS including the efficacy of DO and comparison of DO with other treatment modalities. Since 2018 till now, scholars have gradually embarked on research into the use of DO in the treatment of other craniomaxillofacial disorders such as obstructive sleep apnea, TMJ ankylosis and cleft lip and palate.

**Figure 6:**
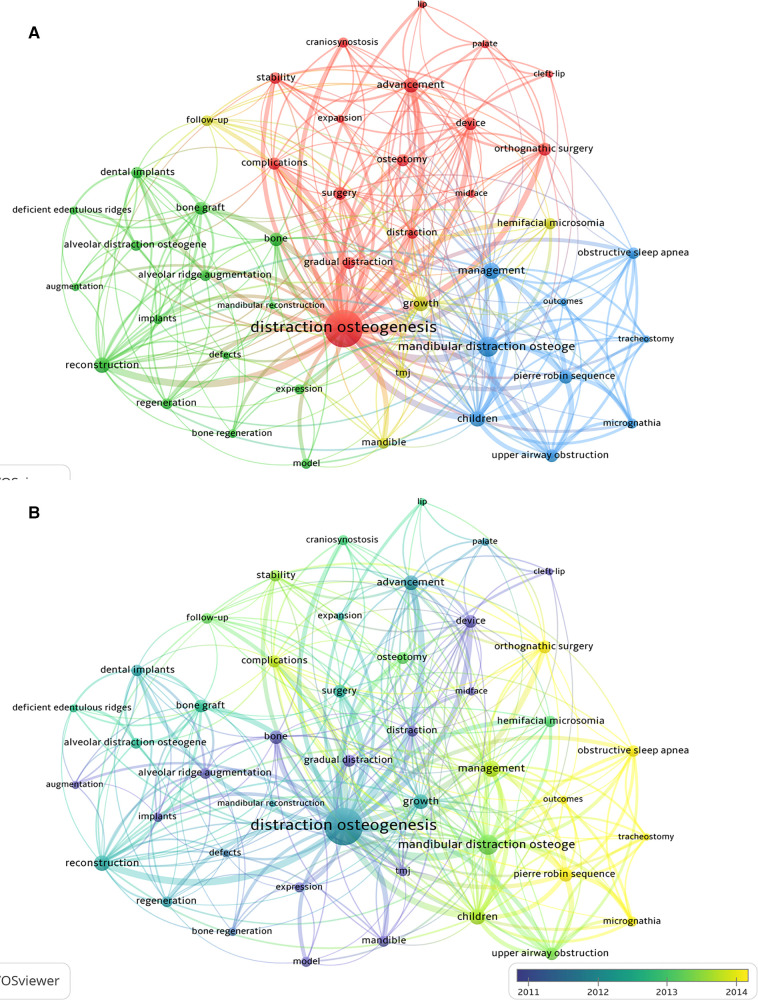
The co-occurrence network of keywords related to research on craniomaxillofacial distraction osteogenesis from 2000 to 2021. (**A**) Clustering analysis of keywords; (**B**) Timeline view of keywords with frequency no less than 80.

The clustering analysis showed that the keywords with high frequency were grouped into three primary clusters (Figure 6A). The green cluster was mainly associated with maxillofacial defect repair and dental implant-related alveolar bone augmentation, including “reconstruction”, “alveolar distraction augmentation”, “bone graft”, “dental implant” and so on. The red cluster focuses on application methods, application sites and the advancement, including “advancement”, “surgery”, “complications”, “gradual distraction”, “osteotomy”, “craniosynostosis” and so on. The blue cluster mainly focused on syndromes resulting from craniomaxillofacial developmental defects, including “mandibular distraction osteoge”, “children”, “management”, “pierre robin sequence”, “upper airway obstruction”, “obstructive sleep apnea” and so on.

The timeline view of the keywords with high frequency was provided in Figure 6B. The studies related to the principle of DO and its application in oral implantology and maxillofacial deficiency were carried out earlier, while in the last few years, the research hotspots have focused on its application in orthognathic surgery and the treatment of PRS, obstructive sleep apnea and other craniomaxillofacial disorders.

## Discussion

We conducted a bibliometric analysis of publications on maxillofacial distraction osteogenesis from 2000 to 2021. The number of annual publications and citations increased significantly, according to the results. The United States, the People's Republic of China and Italy are the most dominant forces in research related to this topic. Five of the top ten most productive institutions were located in the United States, four in China, and one in Italy. Although only one institution from Italy is listed, two institutions, University of Milan and University of Bologna, from Italy have the highest number of citations, indicating the high quality of their publications. Buchman SR is the most productive author, while McCarthy JG is the most influential author.

The most influential journal is *Plastic and Reconstructive Surgery*, whereas the most prolific journal is *the Journal of Oral and Maxillofacial Surgery*.

### References with burst

The top 3 most recent references with burst are all related to micrognathia, specifically PRS. Breik et al. (16) published “*Mandibular distraction osteogenesis for the management of upper airway obstruction in children with micrognathia: a systematic review*” in 2016, which found that mandibular distraction osteogenesis (MDO) was highly successful (95.5 percent success rate) in preventing tracheotomies in children with micrognathia who had poor conservative treatment results. MDO had a lower success rate (81 percent) in facilitating decannulation of children depending on tracheotomy. The most common causes of failure are severe preoperative gastro-oesophageal reflux disease, swallowing dysfunction and tracheotomy-related complications. The long-term complications caused by MDO need to be further investigated. Breugem et al. (17) published “*Best Practices for the Diagnosis and Evaluation of Infants With Robin Sequence: A Clinical Consensus Report.*” in 2016 and provided agreed-on recommendations for clinical features and evaluations of Robin Sequence(RS). The diagnostic points of RS are micrognathia, glossoptosis and airway obstruction. “*Airway and Feeding Outcomes of Mandibular Distraction, Tongue-Lip Adhesion, and Conservative Management in Pierre Robin Sequence: A Prospective Study.*” by Khansa et al. (18) in 2017 evaluated three approaches to the treatment of PRS in 2017: MDO, tongue-lip adhesion, and conservative management. The results suggested that conservative treatment was best suited to neonates with a stable airway in the side or prone position, who can tolerate oral feeding and gain weight, that tongue-Lip adhesion was best suited to neonates with a less stable airway or poor oral intake and an intermediate apnoea-hypopnoea index, and that MDO was suitable for neonates with a severely compromised airway and a severely underdeveloped mandible with a high apnoea-hypopnoea index.

### Keywords with burst

In recent years, three keywords with burst related to craniomaxillofacial DO have emerged: obstructive sleep apnea, TMJ ankylosis, and cleft lip and palate.

#### Obstructive sleep apnea

The most cited studies related to obstructive sleep apnea focus on the use of MDO in treatment of pediatric micrognathia such as PRS, bilateral hemifacial microsomia (due to mandibular growth disturbance), Treacher Collins syndrome, Nager syndrome and so on (19). MDO gradually lengthens the mandible allowing the tongue and supraglottis to move forward (20), effectively increasing airway space in pediatric patients with micrognathia and airway obstruction, improving their airway obstruction, preventing tracheotomy and facilitating extubation during tracheotomy (21–23). Failure to extubate after MDO can be attributed to complications of the retraction process or inherent anatomical airway defects that are not detectable beforehand (20), such as the relatively low success rate of MDO-facilitated extubation in tracheotomized patients with complex congenital syndromes (24). However, in these publications with burst, there is no definitive evidence or clear recommendations regarding the osteotomy designs, the determination of whether a latency period is required, or the assessment of long-term stability, and there is no consensus on the precise indications and timing of MDO surgery (20).

There are some articles related to the use of distraction osteogenesis maxillary expansion (DOME) in midfacial dysostosis. Premature closure of the skull sutures, as seen in Crouzon, Apert, and Pfeiffer syndromes, causes midface recession and different sleep-related breathing disorders due to velopharyngeal obstruction in young patients and adults (20). DOME can drive the midface forward, but there is less evidence for the efficacy of this approach in airway obstruction (19).

Recent related research has focused on the specific mechanisms and improved procedures of DO for the treatment of OSA. MDO is widely thought to increase airway space by gradually lengthening the mandible, allowing the tongue and supraglottis to move forward, with studies focusing on the specific mechanism of the change in airway morphology (20). A recent analysis based on computational fluid dynamics has revealed that DO may be effective in treating OSA by widening the upper airway and reducing inspiratory resistance (25). In advancement of midface, the magnitude of horizontal advancement correlates closely with the increase in airway volume and improvement in the apnea hypoventilation index (26). Regarding the modified DO procedure for the treatment of OSA, Counterclockwise craniomaxillofacial distraction osteogenesis (C3DO) is indicated for patients with complex and severe syndromes such as maxillary defects, high occlusal plane, and severe airway obstruction (27). The facial skeletal deformity and expansion of upper airway capacity can be addressed by turning the subcranial complex *en bloc* around the nasofrontal junction (27). A variation of the previously performed Le Fort III distraction surgery, Le Fort II distraction with zygomatic relocation, allows for a clockwise rotation of the jaws while advancing the midface horizontally (26) to correct the patient's abnormal facial proportions (28, 29). Rare complications following DO, such as retractor breakage, have also received attention (30).

#### TMJ ankylosis

A few successful cases of MDO reconstruction of the TMJ in patients with TMJ ankylosis have been reported in studies. Compared to autologous grafts, MDO allows for shorter hospital admissions and operation times, reduces surgical risks and recurrence rates, and allows the patient to perform jaw movements including opening and closing the mouth and chewing during the process of bone production (31, 32). MDO can be used alone to reconstruct the TMJ or in combination with arthroplasty (33, 34). External distractions are used in most cases, but intraoral distraction osteogenesis followed by arthroplasty, in conjunction with physiotherapy, has been used to successfully treat TMJ ankylosis in combination with endoscopic techniques (35). A recent study performed a finite element analysis of intraoral and extraoral distraction devices and found that an internal device provided better bone protection and reduced stress on the mandible, whereas an external device allowed for greater traction length, making the use of an intraoral device more recommended (36). However, there was no difference in assessing quality of life (QoL) when utilizing an exterior or internal distractor (37). In terms of recently applied modifications, reverse sagittal split can be used when it is not an option for the treatment of dentofacial malocclusion after gap arthroplasty due to poor proximal control, concerns about poor split and financial constraints (38), bidirectional distractors and concomitant neocallous moulding (CM) can be used in high-angle facial types to prevent an anterior open bite (39) and bring about better Qol results (37), and the placement of microimplants in the jaw bones can also help control the direction of distraction (40).

#### Cleft lip and palate

In the most referenced CLP research, the efficacy of DOME in treating severe maxillary hypoplasia in patients with cleft lip and palate (CLP) was assessed (41, 42). In contrast to DO, conventional osteotomy for the treatment of deformities in CLP patients with severe maxillary hypoplasia often require simultaneous mandibular osteotomy, which affects the aesthetics of the lower face (42, 43). The anterior displacement of the upper jaw may also have an adverse effect on pronunciation, and traditional osteotomy has a high recurrence rate (42, 43). DOME using adjustable rigid external distraction (RED) allows predictable control of the distraction process. Greater maxillary advancement is obtained (42) and the technique is relatively simple, requiring no bone graft or rigid fixation hardware and providing better long-term stability (41, 44). The interdental DO technique can effectively reduce alveolar clefts/fistulas and reconstruct alveolar defects in CLP patients with alveolar defects (45). This is performed by distracting and transporting the distal section of the osteotomized dental arch towards the cleft or defect using a toothborne intraoral distraction device (45). However, there is still a lack of studies that evaluate the efficacy of treatment in terms of changes in the soft to hard tissue ratio in 3D imaging.

Recently, researchers have focused on the molecular mechanism of DOME in the treatment of cleft lip and palate, suggesting that fibroblasts in the zygomatic maxillary suture transform into a vascular endothelium-like state in response to mechanical stimulation, promoting angiogenesis under tension, while secreting signal molecules to interact with other cell types and promote osteogenesis (46, 47). Further research is needed, however, on the mechanistic immune status of craniofacial sutures and the interactions between immune cells and other cell types (48). In terms of improved technology, 3D virtual surgical planning (VSP) and CAD/CAM technology can effectively reduce the total treatment time and allow patients to obtain a class I occlusion at the conclusion of the distraction (48) and the The use of a tooth-borne intraoral device in conjunction with the Mid-Maxillary Osteodistraction (MMOD) technique has been shown to be effective (49). However, the long-term stability of these improved techniques remains to be investigated. Furthermore, the importance of presurgical education for patients undergoing RED for maxillary advancement has been highlighted, and it has been discovered that presurgical education can significantly reduce the length of hospital stay and narcotic pain medication use (50).

### Limitations

There are still some limitations to this study. Firstly, all data for this study are solely acquired from WOSCC. Then, due to the lack of technical approach to combine data from different literature databases in CiteSpace and VOSviewer, and language restrictions in the WOSCC database, only English publications were obtained through literature search. Finally, due to technical defects of the CiteSpace and VOSviewer as the bibliometric software, self-citation could not be eliminated, potentially resulting in bias. However, as far as we are aware, this study is the first bibliometric analysis to focus on craniomaxillofacial DO and provides relevant researchers with a comprehensive insight of the trends and current status of craniomaxillofacial DO research.

## Conclusion

Based on the bibliometric and visual analysis of the research on craniomaxillofacial DO from 2000 to 2021, the findings can be summarized as follows:

(1) The number of publications on craniomaxillofacial DO has increased significantly since 2000 till now, and there was a boost increase in annual citation times in 2021.
(2) The United States, the People's Republic of China, and Italy produce the great majority of publications. Of all the institutions, University of Milan and University of Bologna are the most influential in this field. McCarthy JG is the most influential author.
(3) The research hotspots have shifted from maxillofacial defect repair and dental implant-related alveolar bone augmentation to syndromes resulting from craniomaxillofacial developmental defects. Obstructive sleep apnea, TMJ ankylosis and cleft lip and palate are potential research direction in this field. Future research should concentrate on the precise indications and timing of craniomaxillofacial DO, as well as the long-term outcomes of various modified procedures.

## Data Availability

The original contributions presented in the study are included in the article/**Supplementary Material**, further inquiries can be directed to the corresponding author/s.
