# Supplementary material for: A bibliometric analysis of research on craniomaxillofacial distraction osteogenesis from 2000 to 2021
Source: Front Surg. 2022 Aug 1;9:932164. doi: 10.3389/fsurg.2022.932164 (PMC9377540; doi:10.3389/fsurg.2022.932164)
Supplement: Supplementary file 1 [file Table_1_v1.docx]

| No. | Corrections | Content modification | Result |
| --- | --- | --- | --- |
| 1 | Zheng, L. W., Ma, L. & Cheung, L. K. Comparison of gene expression of osteogenic factors between continuous and intermittent distraction osteogenesis in rabbit mandibular lengthening (vol 108, pg 496, 2009). *ORAL SURGERY ORAL MEDICINE ORAL PATHOLOGY ORAL RADIOLOGY AND ENDODONTOLOGY* **111**, 668-668, doi:10.1016/j.tripleo.2011.03.001 (2011). | The project number for the AO Research Fund was incorrectly reported as “S-07-14Z.” The correct project number is “S-07-76Z.” The correct statement of support should read “Project no. S-07-76Z was supported by the AO Research Fund of the AO foundation. | Republished |
| 2 | Shibuya, Y. Prevention of Lingual Inclination of the Transport Segment in Vertical Distraction Osteogenesis in the Mandible (vol 21, pg 374, 2012). *IMPLANT DENTISTRY* **22**, 202-202, doi:10.1097/ID.0b013e31828ba644 (2013). | The first author’s name was incorrect. The author’s name should have appeared as Yasuyuki Shibuya, DDS, PhD. | Republished |
| 3 | Satoh, K., Suzuki, T., Uemura, T. & Hosaka, Y. Maxillo-mandibular distraction osteogenesis for hemifacial microsomia in children (vol 49, pg 572, 2002). *Annals of plastic surgery* **50**, 303-303 (2003). | The second author of the above-mentioned article was erroneously listed as Takayuki Suzuki, MD.  The author's correct name is Hiroyuki Suzuki, MD. | Republished |
| 4 | Sant'Anna, E. F. *et al.* Combined maxillary and mandibular distraction osteogenesis in patients with hemifacial microsomia (vol 147, pg 566, 2015). *AMERICAN JOURNAL OF ORTHODONTICS AND DENTOFACIAL ORTHOPEDICS* **148**, 11-11 (2015). | The image labeled 9C was wrong. Here is the correct version of Figure 9. 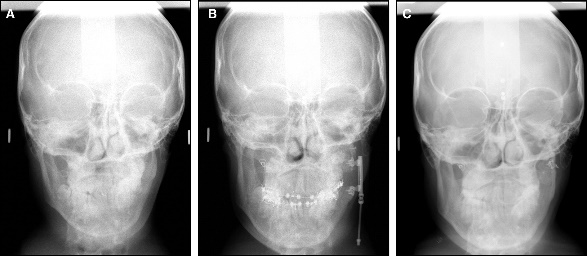 | Republished |
| 5 | Sant'Anna, E. F. *et al.* Micro-computed tomography evaluation of the glenoid fossa and mandibular condyle bone after bilateral vertical ramus mandibular distraction in a canine model (vol 17, pg 111, 2006). *JOURNAL OF CRANIOFACIAL SURGERY* **17**, 611-619, doi:10.1097/00001665-200605000-00041 (2006). | 1. MATERIALS AND METHODS section:   The original statement is ”The dogs received ampicillin, 250 mg/mL, for antibiotic coverage and buprenorphine, 0.3 mg/mL, twice a day for 5 days postoperatively to minimize discomfort or pain. After a latency period of 7 days, distraction was initiated at a rate of 1 mm/d (0.5 mm every 12 hours). The animals were divided into two experimental groups (N = 4) and killed under anesthesia with intravenous KCL (60 mg/kg) after a 30- or60-day period of consolidation.”  The correct statement is “The dogs received intramuscular ampicillin, 250 mg/mL, twice a day for five days and antibiotic coverage and buprenorphine, 0.3 mg/mL, twice a day for 5 days postoperatively to minimize discomfort or pain. After a latency period of 7 days, distraction was initiated at a rate of 1 mm/d (0.5 mm every 12 hours) for 12 consecutive days. The animals were divided into two experimental groups (N = 4) and killed under anesthesia with intravenous KCL (5 mL; 0.3 mg/mL) after a 30- or 60-day period of consolidation.”  2. Figure 4 and Figure 5 are reproduced correctly.  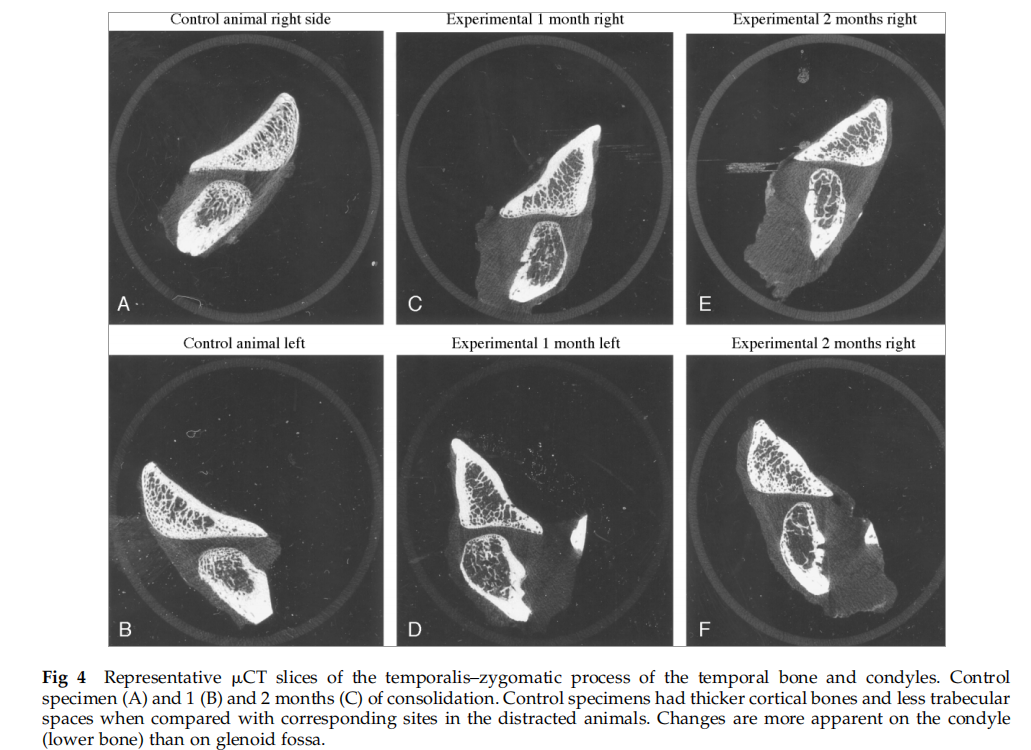 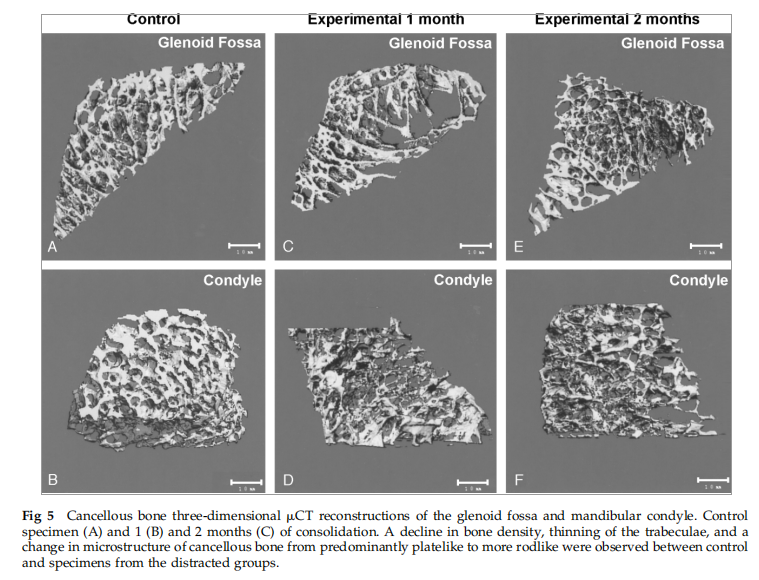   1. DISCUSSION section:   The original statement is “Such a trabecular structure is optimally adapted to sustain loads in directions that coincide with the majority of joint forces applied to the condyle during mastication.”  The correct statement is “Such a trabecular structure is optimally adapted to sustain loads in directions that coincide with the majority of joint forces applied to the condyle during mastication and also to supply nutrition to the avascular cartilage.” | Republished |
| 6 | Menezes, D. J. B., Shibli, J. A., Gehrke, S. A., Beder, A. M. & Sendyk, W. R. Effect of platelet-rich plasma in alveolar distraction osteogenesis: a controlled clinical trial (vol 54, pg 83, 2016). *BRITISH JOURNAL OF ORAL & MAXILLOFACIAL SURGERY* **55**, 447-447, doi:10.1016/j.bjoms.2016.02.003 (2017). | The authors regret < that the dosage of amoxicillin was unclear in the original version of the article. The correct dosage is ‘2 capsules of amoxicillin 875 mg orally’ instead of ‘2 g amoxicillin 875 mg orally’>. | Republished |
| 7 | Laster, Z., Rachmiel, A. & Jensen, O. T. Alveolar width distraction osteogenesis for early implant placement (vol 63, pg 1724, 2005). *JOURNAL OF ORAL AND MAXILLOFACIAL SURGERY* **64**, 566-566 (2006). | The title for Adi Rachmiel was listed incorrectly. The correct information for all authors appears below:  Zvi Laster, DDS, Professor and Director, Department of Oral and Maxillofacial Surgery, Poriya Hospital, Poriya, Israel.  Adi Rachmiel, DMD, PhD, Deputy Head of Department and Adjunct Senior Lecturer, Department of Oral and Maxillofacial Surgery, Rambam Medical Center and the Bruce Rappaport Faculty of Medicine, Technion-Israel Institute of Technology, Haifa, Israel.  Ole T. Jensen, DDS, MS, Private Practice, Denver, CO. | Republished |
| 8 | Kessler, P., Wiltfang, J., Schultze-Mosgau, S., Hirschfelder, U. & Neukam, F. W. Distraction osteogenesis of the maxilla and midface using a subcutaneous device: report of four cases (vol 39, pg 13, 2001). *BRITISH JOURNAL OF ORAL & MAXILLOFACIAL SURGERY* **39**, 165-165 (2001). | The publishers wish to apologize for the fact that, on page 18 of this paper, Figure 1B was repeated in place of the correct Figure 4B.  Figures 1 and 4 are reproduced correctly below with the relevant captions.  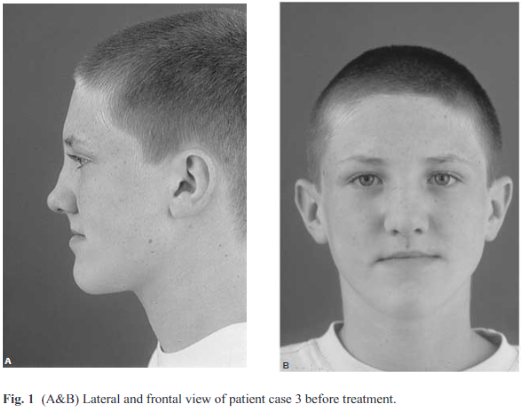  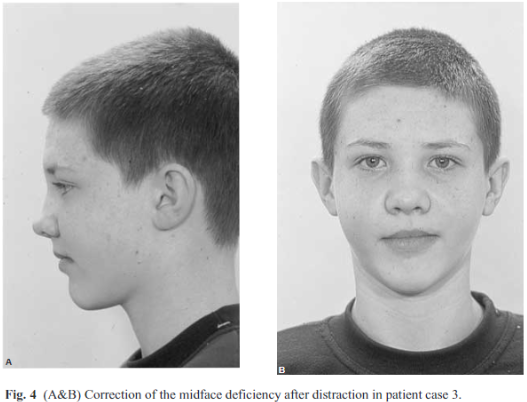 | Republished |
| 9 | Ginini, J. G. *et al.* Effects of Extracorporeal Shock Wave Therapy on Distraction Osteogenesis in Rat Mandible (vol 142, pg 1501, 2018). *PLASTIC AND RECONSTRUCTIVE SURGERY* **143**, 654-654, doi:10.1097/PRS.0000000000005585 (2019). | The second paragraph of the article’s Material and Methods section was missing the citation to their additional article on the same population of animals which completes the current work: “Ginini JG, Emodi O, Sabo E, Maor G, Shilo D, Rachmiel A. Effects of Timing of Extracorporeal Shock Wave Therapy on Mandibular Distraction Osteogenesis: An Experimental Study in a Rat Model. J Oral Maxillofac Surg. | Republished |
